# Use of public data to describe COVID-19 contact tracing in Hubei Province and non-Hubei provinces in China between 20 January and 29 February 2020

**DOI:** 10.5365/wpsar.2021.12.3.808

**Published:** 2019-08-12

**Authors:** Emilio Dirlikov, Suizan Zhou, Lifeng Han, Zhijun Li, Ling Hao, Alexander J. Millman, Barbara Marston

**Affiliations:** a United States Centers for Disease Control and Prevention, Atlanta, GA, United States of America.; b United States Centers for Disease Control and Prevention, China Country Office, Beijing, China.; c United States Public Health Service Commissioned Corps, Rockville, MD, United States of America.

## Abstract

**Objective:**

Contact tracing has been used in China and several other countries in the WHO Western Pacific Region as part of the COVID-19 response. We describe COVID-19 cases and the number of contacts traced and quarantined per case as part of COVID-19 emergency public health response activities in China.

**Methods:**

We abstracted publicly available, online aggregated data published in daily COVID-19 situational reports by China’s National Health Commission and provincial health commissions between 20 January and 29 February 2020. The number of new contacts traced by report date was computed as the difference between total contacts traced in consecutive reports. A proxy for the number of contacts traced per case was computed as the number of new contacts traced divided by the number of new cases.

**Results:**

During the study period, China reported 80 968 new COVID-19 cases and 659 899 contacts. In Hubei Province, there were 67 608 cases and 264 878 contacts, representing 83% and 40% of the total, respectively. Non-Hubei provinces reported tracing 1.5 times more contacts than Hubei Province; the weekly number of contacts traced per case was also higher in non-Hubei provinces than in Hubei Province and increased from 17.2 in epidemiological week 4 to 115.7 in epidemiological week 9.

**Discussion:**

More contacts per case were reported from areas and periods with lower COVID-19 case counts. With other non-pharmaceutical interventions used in China, contact tracing and quarantining large numbers of potentially infected contacts probably contributed to reducing SARS-CoV-2 transmission.

Coronavirus disease 2019 (COVID-19) is a respiratory illness caused by infection with severe acute respiratory syndrome coronavirus 2 (SARS-CoV-2), first identified in December 2019 in Hubei Province, China. (*1*) By 31 January 2020, at least one case had been reported from each of mainland China’s 31 provincial-level administrative units, and by 29 February, a total of 80 968 cases had been reported. (*2*) On 30 January 2020, WHO declared COVID-19 a public health emergency of international concern, and on 11 March 2020, WHO declared the outbreak a global pandemic.

Non-pharmaceutical interventions (NPIs) for respiratory virus outbreaks are used to prevent exposures and reduce transmission through individual or community action. (*3*, *4*) With several other countries in the Western Pacific Region, (*5*-*7*) China implemented COVID-19 contact tracing with quarantine as part of a comprehensive COVID-19 prevention and control strategy, which also included mask use, emphasis on hand hygiene, enforced physical distancing and movement restrictions within and between provinces. (*8*, *9*)

China’s contact-tracing strategy was to identify and quarantine exposed individuals to prevent additional disease transmission. On 20 January 2020, China designated COVID-19 a notifiable disease and updated the “Frontier Health and Quarantine Law” to allow quarantine of contacts. (*10*) National guidelines on epidemiological investigations and management of contacts were issued and updated several times, and responsibility for contact tracing was delegated to the local level. (*11*, *12*) The national guidelines defined contacts as: “anyone who may have had contact with a case through a range of circumstances or activities including being family members, relatives, friends, colleagues, classmates, health care workers, and services personnel.” (*12*) The national guidelines further detailed eight categories of close contacts (e.g. family members living together, direct caregivers or providers of medical treatment or care services and other people considered by onsite investigators to meet the criteria for a close contact).

To describe the number of contacts traced and quarantined per case as part of COVID-19 emergency public health response activities, we compared data from Hubei Province with those from the 30 other mainland provinces (non-Hubei provinces) reported between 20 January and 29 February 2020. We compared the numbers in Hubei Province with those in non-Hubei provinces because the majority of reported cases occurred in Hubei Province.

## Methods

We abstracted publicly available, online aggregated data reported in daily situational reports at the national level by the NHC and provincial level by provincial health commissions (see **Appendix**: data sources). For epidemiological weeks 4–9 (weeks ending on Saturdays), we collected daily reported data on newly reported cases and total contacts traced and placed under medical observation. Data were reviewed for abstraction errors, including data entry errors and data completeness. Provincial data that were > 95% complete (i.e. reporting for > 95% of days between 20 January and 29 February) were included. When situational reports included corrections to reported data, the corrected data were used for the day reported.

The number of new contacts traced by report date was computed as the difference between the total number of contacts traced on consecutive reports. A proxy for the number of contacts traced per case was computed as the number of new contacts traced divided by the number of new cases. Calculations were performed by epidemiological week. Data were analysed at national and provincial levels (in the included provinces) and for the 30 non-Hubei provinces combined, calculated as the difference between national totals and totals for Hubei Province.

### Ethics statement

This activity was deemed not to be research as defined in United States Government 45 CFR 46.102(l), and institutional review board approval was not required. Non-research determination was provided by the US CDC Center for Global Health in May 2020.

## Results

**Figure 1:**
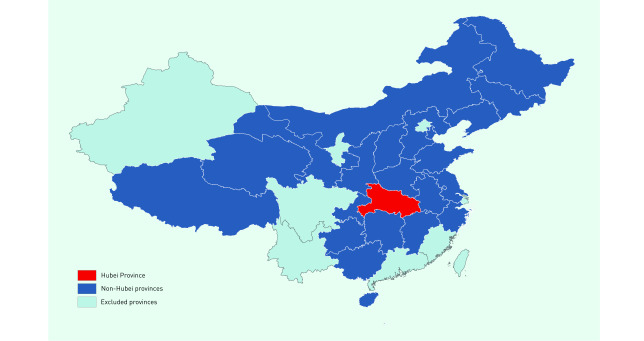
Map of mainland China provinces included in the analysis

In addition to national and Hubei Province data, complete data were available for 22 of 30 non-Hubei provincial-level administrative units: Anhui, Chongqing Municipality, Gansu, Guangxi Autonomous Region, Guizhou, Hainan, Hebei, Heilongjiang, Henan, Hunan, Inner Mongolia Autonomous Region, Jiangsu, Jiangxi, Jilin, Liaoning, Qinghai, Shaanxi, Shandong, Shanxi, Tianjin Municipality, Tibet Autonomous Region and Zhejiang (**Fig. 1**). Eight provinces, comprising 26% of the total population, were excluded from the analysis because of no or insufficient reported data (Beijing Municipality, Fujian, Guangdong, Ningxia Autonomous Region, Shanghai Municipality, Sichuan, Xinjiang Autonomous Region and Yunnan).

[**insert** Figure 1]

During epidemiological weeks 4–9, the NHC reported 80 968 new COVID-19 cases and 659 899 contacts traced. These included 67 608 cases (83% of total cases reported) and 264 878 contacts (40% of total reported contacts traced) in Hubei Province. During the same period, non-Hubei provinces reported an aggregate total of 13 360 cases and 395 021 contacts traced. Among the 22 provinces with provincial-level data, those with the largest numbers of reported cases and contacts traced were Henan Province (reported cases = 1274/9664 [13%]; reported contacts = 39 199/306 684 [13%]) and Zhejiang Province (reported cases = 1216/9664 [13%]; reported contacts = 41 050/306 684 [13%]).

**Figure 2:**
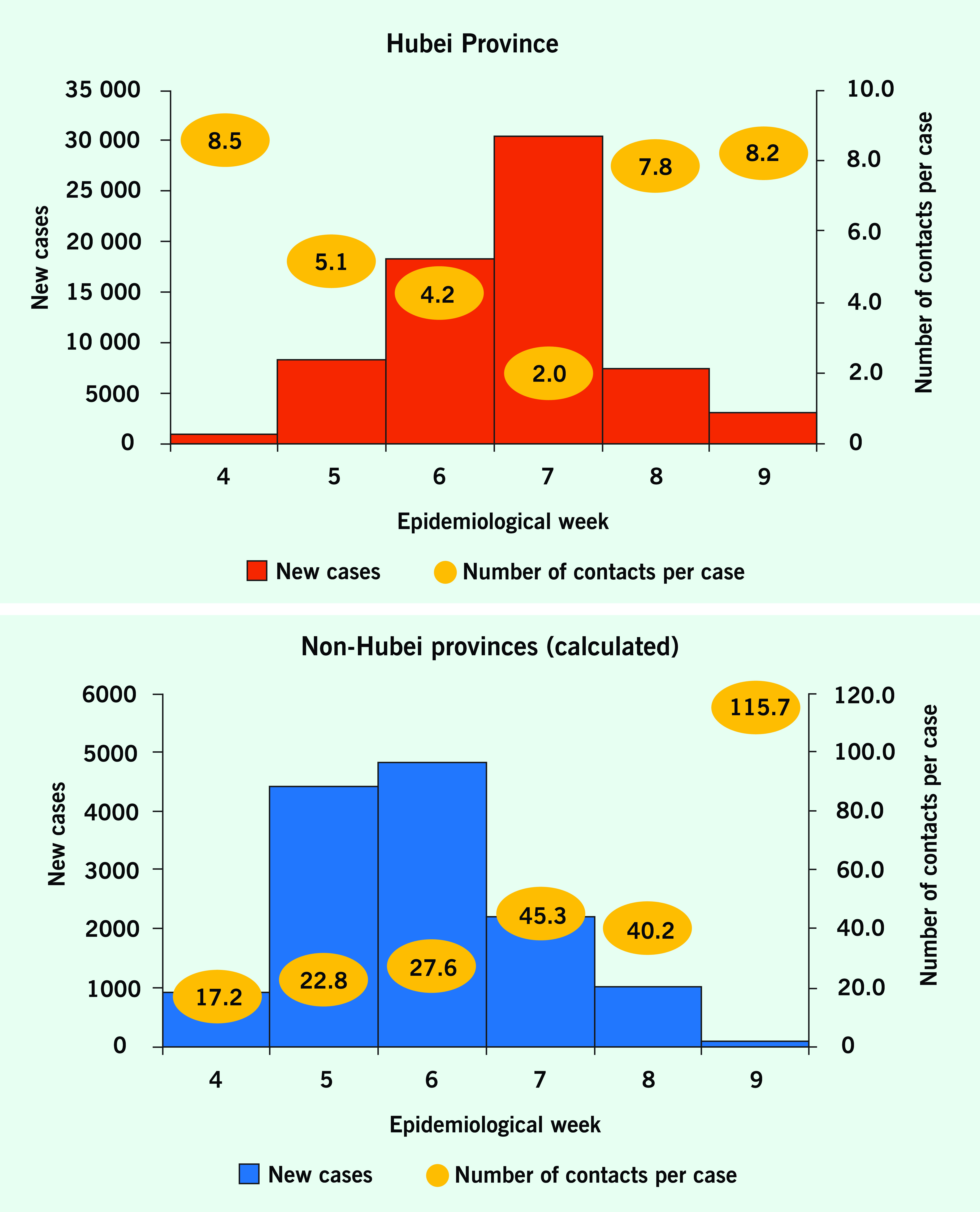
Reported numbers of COVID-19 cases and contacts traced per case, by week, Hubei Province (red) and non-Hubei provinces (calculated; blue), epidemiological weeks 4–9, 2020

The weekly number of contacts traced per case remained < 10 in Hubei Province throughout the study period (median = 6.45; range = 2.0 in epidemiological week 7 to 8.5 in epidemiological week 4); the lowest value occurred when 18 453 clinically diagnosed cases were reported as part of the case counts for 12–15 February (epidemiological week 7), which increased the denominator substantially and consequently reduced the number of contacts traced per case (**Fig. 2** and **Table 1**).

[**insert** Figure 2]

**Table 1:** Weekly numbers of reported COVID-19 cases, contacts traced and contacts traced per COVID-19 case, by geographical unit, epidemiological weeks 4–9, 2020

Epi week	National total			Hubei Province			Non-Hubei provinces (calculated)		
	Cases	Contacts	Contacts per case	Cases	Contacts	Contacts per case	Cases	Contacts	Contacts per case
Epi week 4	1748	22 614	12.9	854	7250	8.5	894	15 364	17.2
Epi week 5	12 410	140 413	11.3	8022	40 582	5.1	4388	99 831	22.8
Epi week 6	22 843	208 061	9.1	18 026	75 256	4.2	4817	132 805	27.6
Epi week 7	32 447	157 513	4.9	30 279	59 356	2.0	2168	98 157	45.3
Epi week 8	8437	99 099	11.7	7409	57 754	7.8	1028	41 345	40.2
Epi week 9	3083	32 199	10.4	3018	24 680	8.2	65	7519	115.7
Total	80 968	659 899	8.2	67 608	264 878	3.9	13 360	395 021	29.6

A proxy for the number of contacts traced per case was computed as the number of new contacts traced divided by the number of new cases. Data for the 30 non-Hubei provinces were calculated as the difference between national totals and totals for Hubei Province.

In Hubei Province, the lowest value occurred when 18 453 clinically diagnosed cases were reported in case counts for 12–15 February (epidemiological week 7), which increased the denominator substantially and consequently lowered the number of contacts traced per case.

The weekly number of contacts traced per case was higher in non-Hubei provinces than in Hubei Province and increased from 17.2 in epidemiological week 4 to 115.7 in epidemiological week 9 (**Fig. 2** and **Table 1**). Data from the 22 non-Hubei provinces indicated that the number of contacts traced per case generally increased as case counts declined, while the reported number of contacts traced either remained high or increased over time. For example, Anhui Province reported 60 cases and 1023 contacts traced during epidemiological week 4 (17.1 contacts traced per case) and 1 case and 915 reported contacts traced during epidemiological week 9 (915 contacts traced per case).

## Discussion

With other NPIs used in China, contact tracing and quarantining of a large number of potentially infected contacts probably contributed to reducing SARS-CoV-2 transmission. (*10*) Contact tracing with quarantine potentially helped to mitigate the risk of transmission by identifying pre-symptomatic and asymptomatic infections early and reducing the time from symptom onset to initiation of medical care. (*13*, *14*)

Contact tracing and data reporting varied by province, with non-Hubei provinces reporting more contacts traced per case, and the number of contacts traced per case in these provinces increasing during the study period. In Hubei Province, the average number of contacts traced per case remained < 10 during this period, and the number of contacts traced decreased with increasing numbers of reported cases. Although non-Hubei provinces reported only 17% of total cases, 1.5 times more contacts were traced than in Hubei Province.

The differences between provinces may reflect local capacity for contact tracing, differences in local disease transmission, evolving guidelines and implementation of other NPIs. For example, a lockdown in Wuhan City began on 23 January 2020, followed by widespread movement restrictions within and between provinces (*1*) to mitigate transmission; national travel restrictions began to be lifted on 17 February 2020, although movement restrictions continued. Implementation differed among provinces. (*9*)

Contact tracing with quarantine is resource intensive. For example, in Wuhan City, contact tracing was conducted by 1800 epidemiologists working in teams of five. (*8*) Data on provincial contact-tracing resources were not available. Geographical and temporal differences may have affected the availability of resources, including trained staff for contact tracing and medical observation, housing for contacts and laboratory testing capacity. While contact tracing identified and isolated large numbers of potentially infected contacts, published studies show that most contacts did not become reported cases: 30.4% (391 positive contacts/1286 contacts traced) in Shenzhen, 2.6% (129/4950) in Guangzhou and 2.3% (120/5241) in Xi’an. (*13*-*15*)

This report has several limitations. First, the public data did not include contact-by-exposure type, and it is likely that the actual number of contacts traced differed by type of exposure (e.g. family, shopping centre, public transport). Therefore, the number of “contacts traced per case” may be overestimated when large numbers of contacts are linked to a single case (e.g. attending a public gathering with a confirmed case). Second, without data on individual patients, our analysis is based on aggregated data and subject to ecological fallacy. For example, contacts traced reported in one week could have been those of cases reported in the previous week. Third, data on contact-tracing outcomes and resources were not available for analysis. All contacts were assumed to have been quarantined according to the national guidelines, and provinces were assumed to have implemented contact-tracing guidelines uniformly, although inter-provincial differences may have affected the comparability of the reported data. The data could not be verified externally, the data collection methods were unknown, and it was not known whether all reported contacts traced were linked to reported cases. Finally, reported data on contact tracing were missing or incomplete for eight provinces, which were excluded from the analysis.

Despite these limitations, our findings describe contact tracing in China during the COVID-19 response and differences between Hubei Province and non-Hubei provinces based on publicly available data. We found higher rates of contacts traced and quarantined in areas with lower numbers of reported COVID-19 cases, suggesting that contract tracing may have been more comprehensive in areas and periods with lower case counts (non-Hubei provinces); there may also have been differences in other NPIs implemented, including mask use, emphasis on hand hygiene, enforced physical distancing and movement restrictions.

Future investigations should better define the role of COVID-19 contact tracing and quarantine, including timeliness, prioritization of contacts who are more likely to be associated with transmission and the effectiveness of contact tracing in contexts that differ epidemiologically, socially and with respect to resource availability.

## References

[1] Pan A, Liu L, Wang C, Guo H, Hao X, Wang Q, et al. Association of public health interventions with the epidemiology of the COVID-19 outbreak in Wuhan, China. JAMA. 2020 5 19;323(19):1915–23. 10.1001/jama.2020.6130 32275295 PMC7149375

[2] Chinese Center for Disease Control and Prevention. Tracking the epidemic. China CDC Weekly, 7 May 2020. Available from: http://weekly.chinacdc.cn/news/TrackingtheEpidemic2020.htm, accessed 22 May 2020.

[3] Qualls N, Levitt A, Kanade N, Wright-Jegede N, Dopson S, Biggerstaff M, et al.; CDC Community Mitigation Guidelines Work Group. Community mitigation guidelines to prevent pandemic influenza – United States, 2017. MMWR Recomm Rep. 2017 4 21;66(1):1–34. 10.15585/mmwr.rr6601a1 28426646 PMC5837128

[4] Fong MW, Gao H, Wong JY, Xiao J, Shiu EYC, Ryu S, et al. Nonpharmaceutical measures for pandemic influenza in nonhealthcare settings – Social distancing measures. Emerg Infect Dis. 2020 5;26(5):976–84. 10.3201/eid2605.190995 32027585 PMC7181908

[5] Cheng HY, Jian SW, Liu DP, Ng TC, Huang WT, Lin HH; Taiwan COVID-19 Outbreak Investigation Team. Taiwan COVID-19 outbreak investigation team. Contact tracing assessment of COVID-19 transmission dynamics in Taiwan and risk at different exposure periods before and after symptom onset. JAMA Intern Med. 2020 9 1;180(9):1156–63. 10.1001/jamainternmed.2020.2020 32356867 PMC7195694

[6] Ng Y, Li Z, Chua YX, Chaw WL, Zhao Z, Er B, et al. Evaluation of the effectiveness of surveillance and containment measures for the first 100 patients with COVID-19 in Singapore – January 2–February 29, 2020. MMWR Morbid Mortal Wkly Rep. 2020;69(11):307–11. doi:10.15585/mmwr.mm6911e1 pmid:32191691. 10.15585/mmwr.mm6911e1 PMC7739977 32191691

[7] COVID-19 National Emergency Response Center, Epidemiology and Case Management Team, Korea Centers for Disease Control and Prevention. Epidemiology and case management team; Korea Centers for Disease Control and Prevention. Coronavirus Disease-19: Summary of 2,370 contact investigations of the first 30 cases in the Republic of Korea. Osong Public Health Res Perspect. 2020 4;11(2):81–4. 10.24171/j.phrp.2020.11.2.04 32257773 PMC7104686

[8] Report of the WHO–China Joint Mission on Coronavirus Disease 2019 (COVID-19). Geneva: World Health Organization; 2020. Available from: https://www.who.int/docs/default-source/coronaviruse/who-china-joint-mission-on-covid-19-final-report.pdf, accessed 22 April 2020.

[9] Lai S, Ruktanonchai NW, Zhou L, Prosper O, Luo W, Floyd JR, et al. Effect of non-pharmaceutical interventions to contain COVID-19 in China. Nature. 2020 9;585(7825):410–3. 10.1038/s41586-020-2293-x 32365354 PMC7116778

[10] CDC Weekly C; The Novel Coronavirus Pneumonia Emergency Response Epidemiology Team. The epidemiological characteristics of an outbreak of 2019 novel coronavirus diseases (COVID-19) – China, 2020. China CDC Wkly. 2020 2 21;2(8):113–22. 10.46234/ccdcw2020.032 34594836 PMC8392929

[11] Center for Disease Control and Prevention C; Chinese Center For Disease Control And Prevention. Guidelines for COVID-19 epidemiological investigations. China CDC Wkly. 2020 5 8;2(19):327–8. 10.46234/ccdcw2020.083 34594649 PMC8392950

[12] Center for Disease Control and Prevention C; Chinese Center For Disease Control And Prevention. Guidelines for investigation and management of close contacts of COVID-19 cases. China CDC Wkly. 2020 5 8;2(19):329–31. 10.46234/ccdcw2020.084 34594650 PMC8392945

[13] Bi Q, Wu Y, Mei S, Ye C, Zou X, Zhang Z, et al. Epidemiology and transmission of COVID-19 in 391 cases and 1286 of their close contacts in Shenzhen, China: a retrospective cohort study. Lancet Infect Dis. 2020 8;20(8):911–9. 10.1016/S1473-3099(20)30287-5 32353347 PMC7185944

[14] Luo L, Liu D, Liao X, Wu X, Jing Q, Zheng J et al. Modes of contact and risk of transmission in COVID-19 among close contacts. medRxiv [preprint]. 2020. doi:10.1101/2020.03.24.20042606 10.1101/2020.03.24.20042606

[15] Zhang H, Ji Z, Cheng Z, Zeng L, Mi B, Cheng F, et al. Epidemiological characteristics of close contact in Xi’an. J Xi’an Jiaotong Univ Med Sci. 2020:1–7. ISSN:1671–8259/CN:61–1399/R.

